# A general framework for dynamic cortical function: the function-through-biased-oscillations (FBO) hypothesis

**DOI:** 10.3389/fnhum.2015.00352

**Published:** 2015-06-16

**Authors:** Gerwin Schalk

**Affiliations:** ^1^National Center for Adaptive Neurotechnologies, Wadsworth Center, New York State Department of HealthAlbany, NY, USA; ^2^Department of Biomedical Sciences, State University of New YorkAlbany, NY, USA; ^3^Department of Neurology, Albany Medical CollegeAlbany, NY, USA

**Keywords:** oscillations, information routing, communication-through-coherence, gating-by-inhibition, oscillatory modulation

## Abstract

A central goal of neuroscience is to determine how the brain's relatively static anatomy can support dynamic cortical function, i.e., cortical function that varies according to task demands. In pursuit of this goal, scientists have produced a large number of experimental results and established influential conceptual frameworks, in particular communication-through-coherence (CTC) and gating-by-inhibition (GBI), but these data and frameworks have not provided a parsimonious view of the principles that underlie cortical function. Here I synthesize these existing experimental results and the CTC and GBI frameworks, and propose the function-through-biased-oscillations (FBO) hypothesis as a model to understand dynamic cortical function. The FBO hypothesis suggests that oscillatory voltage amplitude is the principal measurement that directly reflects cortical excitability, that asymmetries in voltage amplitude explain a range of brain signal phenomena, and that predictive variations in such asymmetric oscillations provide a simple and general model for information routing that can help to explain dynamic cortical function.

## Introduction

Humans are able to rapidly adapt their behavior based on different task demands. While research over the past decades has shown that the structure of the brain is plastic, such as that shown in rapid changes in dendritic boutons during learning (Moser et al., 1994; Piccioli and Littleton, 2014), the long time scale, typically minutes, for such plastic changes in anatomy cannot readily explain changes in function on the time scale of seconds. In pursuit of the search for potential mechanisms that can support this dynamic nature of the brain, studies have produced a large number of experimental results and two influential conceptual frameworks.

These studies occur at different levels of inquiry that span the microscopic domain (i.e., single-neuron neurophysiology) and the macroscopic domain [e.g., electroencephalography (EEG) or behavioral state]. Single-neuron neurophysiology studies often directly relate different physiological processes. For example, many studies showed that cortical neurons preferentially fire during the trough of neuronal oscillations in different frequency bands, such as the theta (4–8 Hz) or alpha (8–12 Hz) bands (Bragin et al., 1995; Harris et al., 2003; Huxter et al., 2003; Buzsaki and Draguhn, 2004; Klausberger et al., 2004; Lee et al., 2005; Siapas et al., 2005; Jacobs et al., 2007; Lorincz et al., 2009; Fell and Axmacher, 2011; Haegens et al., 2011). This demonstrates that oscillatory activity can dynamically modulate the excitability of local neuronal populations, which appears to be important for explaining dynamic brain function.

Other microscopic or macroscopic studies cannot or do not make explicit statements about particular physiological processes. Rather, they apply mathematical procedures to particular brain signal measurements and report the observed relationship of the resulting brain signal features with a particular behavioral or other measurement. For example, in numerous studies scientists applied specific mathematical techniques (such as the Hilbert transform) to the (usually bandpass-filtered) time-varying brain signal voltage measurements to calculate time-varying estimates of the power or phase of oscillatory activity in a particular frequency band. An increasing number of reports have shown that such power or phase measurements can be related to cortical excitability (e.g., Sauseng et al., 2009 or Canolty et al., 2006 respectively). The results for oscillatory phase in these studies suggest that cortical processing is more likely to occur during a specific phase (usually the trough) of the underlying oscillations [i.e., phase-amplitude coupling (PAC)]. While important problems with present PAC signal analysis approaches and their resulting physiological interpretation have been recognized (Aru et al., 2014), the results of these studies do echo the results of the basic neurophysiology studies described above. At the same time, this seemingly direct link to underlying physiological processes does not exist for (the purely mathematical construct of) oscillatory power. In other words, it is unclear how oscillatory power may mechanistically alter cortical excitability. Furthermore, it is unclear why cortical excitability appears to be related to two mathematically completely independent measurements (power and phase) of oscillatory activity.

The relationship of different brain signal features with each other and with cortical excitability is even less clear for other types of brain signal features. For example, for the past several decades, scientists have studied different types of evoked responses (ERPs) such as the P300 (Chapman and Bragdon, 1964), or different types of slow task-related activity [Bereitschaftspotential (BP, Kornhuber and Deecke, 1965), contingent negative variation (CNV, Walter et al., 1964), or slow cortical potentials (SCPs; Birbaumer et al., 1990; He and Raichle, 2009)]. These electrophysiological signals often receive different names that may depend not only on the filtering technique (e.g., spectral analysis vs. signal averaging), but also on the specific area of study. For example, scientists who study the neural basis of movements may call a slowly developing negative potential preceding movements a Bereitschaftspotential (BP, Kornhuber and Deecke, 1965); scientists who study consciousness may call a similar phenomenon a slow cortical potential (SCP, He and Raichle, 2009); and scientists who study response anticipation may call it contingent negative variation (CNV, Walter et al., 1964). These differing naming conventions persist even though these observations share some apparent similarities (in that they are usually reflected in negative voltage shifts), and even though there are observations that link them to other (e.g., frequency-based) phenomena (Shibasaki et al., 1978; He and Raichle, 2009). Similar comments about naming convention could also be made about the large number of different evoked responses (ERPs) that result from actual or anticipated sensory stimulation [e.g., the P3a and P3b (Polich, 2007)]. Finally, recent advances in the local field potential (LFP) and ECoG literature have revealed a number of additional brain signal features that express the relationship between the phases or amplitudes of oscillatory activity at single or across multiple sites [e.g., phase-phase or amplitude-amplitude coupling (Buzsaki and Wang, 2012; Siegel et al., 2012)]. The functional relevance and generating mechanism for these phenomena are currently still largely unclear.

Nevertheless, there have been some proposals for mechanisms that could explain different types of brain signal features. For example, scientists have tried to explain the generation of ERPs by phase resetting (Sayers et al., 1974; Makeig et al., 2002; Fell et al., 2004; Hanslmayr et al., 2007), additions to ongoing oscillations (Shah et al., 2004; Makinen et al., 2005; Mazaheri and Jensen, 2006), or non-zero baselines (Nikulin et al., 2007; Mazaheri and Jensen, 2008).

Despite these present difficulties in understanding how the brain may support dynamic function of individual neuronal populations, scientists have proposed two influential conceptual frameworks to begin to explain rapid variations in behavior across neuronal populations. The first proposal is the *communication-through coherence* (CTC) hypothesis put forth by Fries (2005). The CTC hypothesis is concerned with the mechanism by which the brain may modulate the functional relationship between one sending and one receiving neuronal population. Specifically, CTC's principal thesis is that function may emerge from anatomy through the brain's ability to optimize information transfer by synchronizing the timing of oscillatory activity at the sending and receiving sites. This hypothesis rests fundamentally on the physiological concept of variable cortical excitability, i.e., neuronal firing occurs preferentially at the trough of oscillatory activity (Klimesch et al., 2007; Lorincz et al., 2009; Haegens et al., 2011). CTC has received support from modeling studies (Akam and Kullmann, 2010, 2012) and experimental results (Saalmann et al., 2012; Roberts et al., 2013). In sum, CTC is fundamentally based on oscillatory phase: it explains variable function of a sending and a receiving neuronal population primarily through the degree of phase synchrony of modulatory oscillatory activity at those populations.

The second proposal is the *gating-by-inhibition* (GBI) hypothesis that was formally articulated by Jensen and Mazaheri (2010). This hypothesis is based on a long history of research by a number of scientists, including Pfurtscheller, Klimesch, Jensen, and others. In contrast to the CTC hypothesis, GBI is fundamentally based on oscillatory power: it suggests that neuronal populations that are not related to the task are functionally inhibited by increased oscillatory power in specific frequency bands, such as the alpha (8–12 Hz) band. How this concept, which is based on oscillatory power, may be related to the CTC hypothesis, which is based on oscillatory phase, is uncertain.

In summary, while existing theories have made important progress, our understanding how the microscopic concept of cortical excitability relates to different types of macroscopic brain signal measurements and in turn to organized behavior still appears to be incomplete. Furthermore, it is currently unclear how oscillatory power and phase may interrelate with each other, and if and how the conceptual frameworks proposed by Fries and Jensen can be reconciled. Primarily because of these important issues, different neural or behavioral domains are usually described by independent sets of relatively narrow scientific explanations, which tends to force scientists in a particular discipline to stay within and to conform to the corresponding set of explanations. This situation presents a roadblock to an improved understanding of the function of the brain.

Here I provide a conceptual framework of cortical function that may help to resolve these important problems by synthesizing existing experimental results and theoretical models into two general principles. The first principle of this framework suggests that cortical excitability of a neuronal population is indexed most directly by the voltage amplitude of oscillatory activity. This leads to the notion that the established findings of the relationship of oscillatory power or phase with cortical excitability are essentially indirect by-products of asymmetrically distributed peak/trough amplitudes (i.e., biased oscillations), and that such biased oscillations may underlie a range of other brain signal phenomena. The second principle embeds biased oscillations in a predictive context, applies the result to populations of neurons, and thereby reconciles and extends the CTC and GBI hypotheses. I will refer to the framework that encompasses these two principles as the function-through-biased-oscillations (FBO) hypothesis throughout this paper.

## The FBO hypothesis

### The first principle: biased oscillations link cortical excitability to a range of brain signal phenomena

**Figure 1:**
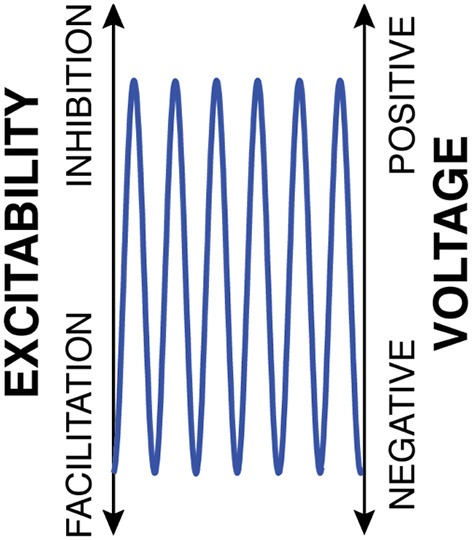
**Oscillatory voltage amplitude is the principal measurement that controls cortical excitability**.

The first principle of the FBO hypothesis begins with the proposal that the instantaneous voltage amplitude of oscillations, rather than oscillatory power or phase, is the principal measurement that directly reflects cortical excitability. Specifically, I suggest that, for the exemplary oscillation shown with the blue trace in Figure 1, the y axis simultaneously represents cortical excitability as well as oscillatory voltage (This exemplary oscillatory activity is shown to be sinusoidal, but in reality may take on different shapes).

**Figure 2:**
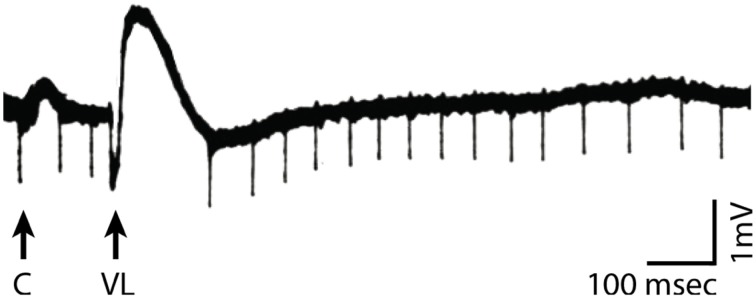
**Recordings in motor cortex close to the cortical surface (black trace) in response to stimulation of the cortex (arrow marked by “C”) or the nucleus ventralis lateralis of the thalamus (arrow marked by “VL”)**. VL stimulation, but not cortical stimulation, results in a change in voltage potential that temporarily suspends motor cortical neuronal firing (Modified from Li, 1956).

Experimental evidence supports this proposed link between changes in instantaneous voltage and cortical excitability. For example, Figure 2 shows recordings from cat motor cortex about 0.2 mm below the cortical surface. Spontaneous firings of motor action potentials are clearly visible. Stimulation of the nucleus ventralis lateralis (i.e., the thalamic nucleus projecting to that area of cortex), but not stimulation of a nearby cortical site, changes the voltage potential and temporarily suspends action potential firing. In other words, thalamocortical volleys appear to shift the cortical voltage potential away from its baseline^1^ so as to hyperpolarize cortical populations and thereby inhibit their firing. Similar effects have been found in the visual cortex (Von Baumgarten and Jung, 1952; Tasaki et al., 1954) and somatosensory cortex (Li et al., 1956). Thus, rhythmically occurring volleys (such as those produced by oscillatory activity) would periodically inhibit a particular neuronal population in the cortex. This resulting interpretation of the functional role of oscillatory activity is consistent with an emerging view on this topic (Klimesch et al., 2007; Mathewson et al., 2011).

It is important to recognize that, in the example in Figure 1 that features a constant and high level of peak-to-peak amplitude, the concepts of oscillatory voltage amplitude and oscillatory phase are essentially interchangeable with respect to their relationship to cortical excitability: excitability is high during a certain phase of the oscillation (i.e., the trough), and excitability is high when the voltage amplitude is low.

**Figure 3:**
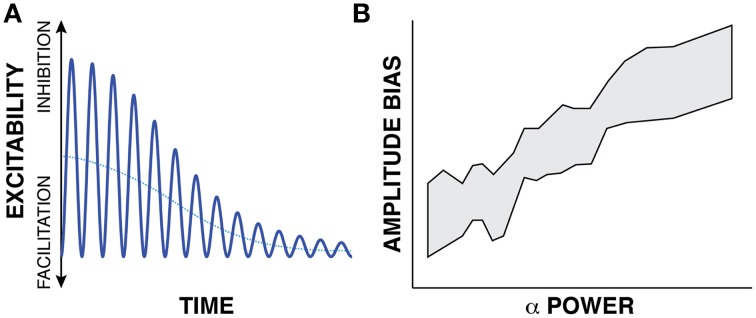
**(A)** The time-varying instantaneous voltage amplitude of oscillatory activity (solid blue trace) is not zero mean, but has a bias (dotted blue trace) whose amplitude varies with the amplitude of oscillatory power. **(B)** Experimental evidence supporting this proposed relationship (Modified from Nikulin et al., 2007).

It is well-known that an oscillation's peak-to-peak amplitude (and hence, oscillatory power) is not constant but often changes with a task. The next building block supporting the first principle of the FBO hypothesis is the suggestion that such task-related changes in peak-to-peak amplitude do not affect the peaks and troughs of the oscillation equally. Let us consider the exemplary oscillatory signal in Figure 3A. In this example, the blue trace gives the time course of oscillatory activity. The peak-to-peak amplitude of this modulatory signal decreases with time (i.e., reduces oscillatory power with time), thereby indicating an overall trend toward increased cortical excitability. As recognized in earlier observations (Nikulin et al., 2007; Mazaheri and Jensen, 2008, 2010) that were made in the context of explaining ERPs, such changes in peak-to-peak amplitude might not affect the amplitude of the peaks and troughs of the oscillatory activity equally, but only affect the amplitude of the peaks^2^. Indeed, Figure 3B (modified from Figure 3A, Nikulin et al., 2007) demonstrates that the amplitude bias of an oscillation in the alpha band (y axis) is related to the power of the oscillation (x axis). (The shaded area gives the 95% confidence interval.) In summary, the second building block of the first principle of the FBO hypothesis suggests that the amplitude bias (dotted blue trace, which could be computed by averaging one cycle of the oscillation or by averaging many trials with random oscillatory phase) is related to oscillatory power.

These two building blocks, i.e., instantaneous voltage amplitude of oscillations reflecting cortical excitability and the existence of a voltage bias, provide the basis for two insights that represent the main conceptual contribution of the first principle of the FBO hypothesis.

The first insight is that the concept of variations in instantaneous voltage amplitude of biased oscillations provides a simpler, more complete, and more physiologically plausible model of cortical excitability than a model based on either oscillatory power or oscillatory phase. It is simpler, because it depends on only one model-free measurement (the instantaneous voltage) rather than on two separate mathematically extracted transformations (power and phase) that depend on a specific model (e.g., a repeating sinusoid).

**Figure 4:**
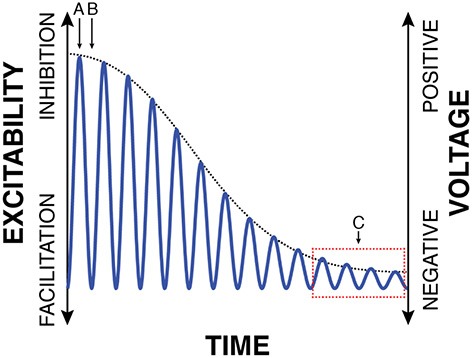
**Traditional interpretations of the relationship between oscillatory power and phase with cortical excitability do not fully capture the realities of biased oscillations**. E.g., oscillatory power is high but cortical excitability is high as well (B); oscillatory phase cycles between peaks and troughs, but cortical excitability is always high (C). In contrast, traditional measurements are correct at (A): oscillatory power is high, oscillatory phase is at a peak, and excitability is low.

This model is also more complete in describing cortical excitability than a model based on either oscillatory power or oscillatory phase. This is apparent in the example in Figure 4. In this example, oscillatory amplitude envelope (dotted black trace, calculated either by using the Hilbert transform or by taking the square root of low-pass filtered oscillatory power) decreases from left to right as the oscillation cycles between different phases of peaks and troughs. Thus, by averaging many measurements, a study may well-find a relationship between oscillatory amplitude/power envelope^3^ and cortical excitability, or between oscillatory phase and cortical excitability, but neither relationship will be entirely correct. Specifically, consider the left-most period of the oscillation in Figure 4. At time (A), oscillatory power accurately reflects cortical excitability: power is high and cortical excitability is low. However, at time (B), there is a big discrepancy between these measurements as power is still high but cortical excitability is high as well. In contrast, for low values of oscillatory power [i.e., around the times indicated by (C)], oscillatory phase cycles between the peak and trough (which would suggest strongly varying cortical excitability), but cortical excitability is relatively constant and high. In contrast, the instantaneous voltage amplitude (that includes the voltage bias) always accurately reflects cortical excitability.

Finally, this model is also more physiologically plausible. As indicated above, several studies have found an inhibitory effect of voltage shifts produced by subcortical volleys on firing of cortical populations (Von Baumgarten and Jung, 1952; Tasaki et al., 1954; Li, 1956; Li et al., 1956). However, such physiological interpretations cannot readily be made for the (purely mathematical concepts of) oscillatory phase or oscillatory power.

**Figure 5:**
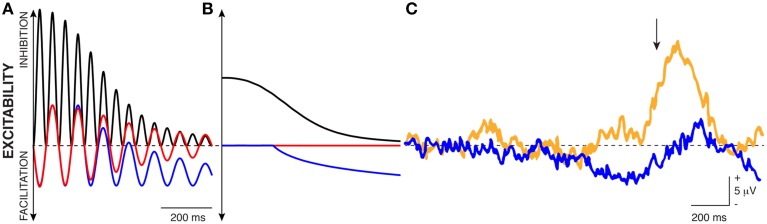
**The bias in oscillations can explain different macroscopic observations. (A)** The blue trace gives the time-varying voltage amplitude of an exemplary biased oscillation. The red trace illustrates the effect of band-pass filtering the blue trace between 8 and 12 Hz. The black trace is the squared amplitude of the red trace. **(B)** Traces show the average of many trials of the corresponding signal traces shown in **(A)** with random phase. **(C)** The blue trace illustrates the average voltage of EEG recordings prior to movement (indicated with an arrow). The yellow trace illustrates the average voltage trace after a lesion to the nucleus ventralis intermedius of the thalamus (Modified from Shibasaki et al., 1978).

The presence of the voltage bias also has important implications for the generating principles of a variety of macroscopic brain signal features. This possibility has been discussed in the specific context of ERPs in previous work (Nikulin et al., 2007; Mazaheri and Jensen, 2008). The second insight is that these implications may be broader than previously discussed. In this context, let us consider the example given in Figure 5. The blue trace in panel A illustrates the time course of the raw (i.e., biased) voltage of an exemplary 10-Hz (i.e., alpha band) modulatory signal in a single trial. Similar to Figure 3A, this exemplary modulatory signal reduces the voltage of its peak over about 1.5 s, thereby indicating time-varying but still progressively increasing cortical excitability. In other words, the instantaneous voltage amplitude of this exemplary blue trace is the result of a 10-Hz oscillation, a slow decrease in peak-to-peak amplitude, and a concomitant decrease in voltage bias. As will become important later, this slow decrease may suggest the physiologically independent presence of a very slow oscillation in a frequency analysis.

There are several ways to extract oscillatory measurements from brain signals (bandpass-filtering, Hilbert transform, etc.). The red trace illustrates the result from subjecting the blue trace to a bandpass filtering operation between 8–12 Hz. Because the bandpass filtering operation removes frequencies lower than 8 Hz, it removes the oscillation's voltage bias: notice how the voltage bias (that is readily visible in the blue trace) disappears in the red trace after the bandpass filtering operation. In other words, the red trace is now centered around zero mean (dashed black line indicating zero voltage). The black solid trace illustrates the instantaneous power (i.e., squared amplitude) of the bandpass-filtered signal.

The blue, red, and black traces in Panel B show the average of many trials of the corresponding oscillatory signal traces shown in Panel A with random phase. The blue average trace highlights a trend toward increasing excitability (i.e., decreasing voltage amplitude), similar to what is usually seen in the BP, SCP, or CNV. The red average trace does not show any variations over time. The black average trace highlights the reductions in oscillatory power typically seen prior to volitional task engagement. Notice the somewhat smoother appearance of the black trace compared to the blue trace, which results from the timing uncertainty introduced by the bandpass filtering operation. In summary, the concept of biased oscillations can explain the relationship between the negative voltage shifts and the decrease in oscillatory power that are often observed in relationship to particular tasks (such as movements).

The literature provides some clues that are consistent with aspects of this hypothesis. One such piece of evidence is shown in Panel C (modified from Shibasaki et al., 1978). The blue trace illustrates the average voltage of EEG recordings prior to movement (indicated with an arrow). The negative deflection prior to movement onset is readily apparent, and is similar to that in the blue trace in Panel B. The yellow trace illustrates the average voltage of EEG recordings after a lesion to the nucleus ventralis intermedius (VIM), i.e., the thalamic nucleus that projects to motor cortex. The yellow trace does not feature the negative deflection prior to movement, but does exhibit an increased ERPs following the movement. In other words, with an intact VIM, we see the typical BP prior to movement. After the VIM has been lesioned, no such negative voltage shift occurs, quite possibly because thalamic lesions often diminish alpha oscillations (Hughes and Crunelli, 2005). In summary, the second insight of the first principle of the FBO hypothesis is that the amplitude bias in oscillatory activity may explain aspects of the slow time-varying brain signal phenomena that usually precede behaviors.

When integrated with other well-known observations, the same concept may also provide a convenient explanation for evoked responses (ERPs) that follow motor movements or sensory stimulation. Specifically, it is well-known that the brain can modulate not only the peak-to-peak amplitude but also the instantaneous phase of ongoing low-frequency oscillations. This phenomenon is termed phase resetting and has previously been suggested to be a contributing factor to ERP generation (Sayers et al., 1974; Makeig et al., 2002; Fell et al., 2004; Hanslmayr et al., 2007; Sauseng et al., 2007). However, in addition to phase resetting, it is also well-known that different task-related areas in the brain are modulated by different oscillations at similar or different frequencies (Jacobs et al., 2007), and that motor movements or sensory stimulation may result in modulation of oscillatory power (Pfurtscheller and Aranibar, 1979; Potes et al., 2014, respectively). All of these known effects will contribute to a time-varying bias in average voltage, and thereby must all provide an important contribution to the generation of ERPs.

Finally, biased oscillations may also explain some of the more recent observations reported in the literature, including particular reports of PAC, phase-phase coupling, or amplitude-amplitude coupling (Siegel et al., 2012). As an example, for the representative data shown in Figure 5A, analyses may identify PAC between the 10-Hz alpha oscillation and the <1 Hz activity change (See Aru et al., 2014, for a more comprehensive discussion of issues with current analyses or their interpretation.).

In summary, the first principle of the FBO hypothesis suggests that the instantaneous voltage amplitude of biased oscillations is the principal measurement that controls cortical excitability, and that it can help to explain a variety of macroscopic brain signal phenomena.

### The second principle: a general framework for dynamic cortical function

The second principle synthesizes and extends the concepts provided in the CTC hypothesis and the GBI framework by embedding the concept of biased oscillations into a predictive context. The result provides a simple and general model for routing of information flow that can explain dynamic cortical function.

Similar to the proposal that biased oscillatory voltage amplitude provide a unifying foundation for explaining experimental results for oscillatory power and phase, control of local cortical excitability with biased oscillations can also provide a unifying foundation for synthesizing CTC and GBI. The proposal is that rather than controlling the phase relationship of oscillations across task-related populations (as proposed by CTC) or oscillatory power of neuronal populations (as proposed by GBI), the brain engages in dynamic task-related processing by controlling the instantaneous voltage amplitude of biased oscillations to predictively inhibit task-unrelated populations or inhibit populations at task-unrelated times.

**Figure 6:**
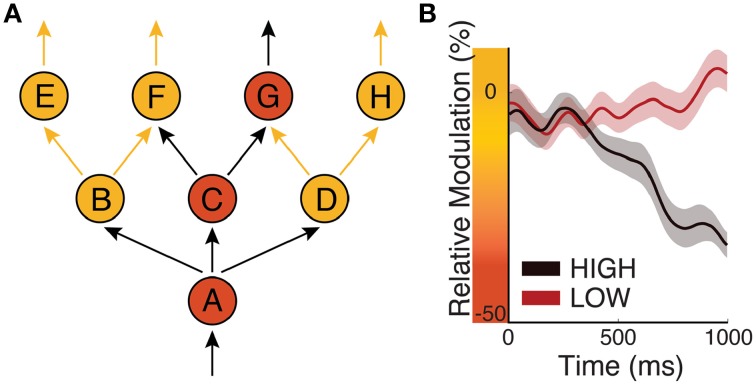
**Biased oscillations regulate information flow in the cortex. (A)** Circles represent eight neuronal populations (A–H). Yellow/orange circles represent populations that receive inhibitory/excitatory input, respectively. Black/yellow arrows represent neuronal pathways that do/do not carry action potentials, respectively. **(B)** Time courses of oscillatory power over sensorimotor cortex in a perceptual decision-making task in which subjects have to push a button depending on sensory evidence. Black/red traces give oscillatory power for “high” or “low” amounts of evidence, respectively (Modified from Kubanek et al., 2013).

To illustrate this concept, let us consider the exemplary network of neuronal populations that is shown in Figure 6A. In this figure, eight distinct neuronal populations are labeled with A–H. Anatomical connections between these populations are depicted with arrows. Arrows that do or do not carry action potential volleys are shown in black or yellow, respectively. Populations that receive excitatory or inhibitory modulation (i.e., low or high average peak-to-peak voltage amplitude, respectively) are shown in orange or yellow, respectively. In this example, population A, which does not receive inhibitory modulation (e.g., from subcortical structures such as a particular thalamic nucleus), receives an action potential volley and sends out volleys to all populations it is connected to (B, C, and D), presumably through cortico-cortical projections. Because B and D receive inhibitory modulation, they are not excited by the incoming volleys they receive from A; thus, they do not send out volleys to connected populations. In this example, excitatory input to population A will result in activation of, and communication between, populations C and G. This concept synthesizes the CTC and GBI hypotheses: because biased oscillatory voltage amplitude can define higher excitability either by decreasing peak-to-peak amplitude or by being in its trough, it can describe a situation in which a sending and a receiving neuronal population communicate either by synchronizing their phases (as would be suggested by CTC) or by decreasing the peak-to-peak amplitude of the receiving population (as would be suggested by GBI).

The second principle anchors the dynamics of biased oscillations in a predictive process. Dynamic information routing may require separate mechanisms for task-related engagement that can or cannot be predicted based on prior evidence. There are obvious situations in which our interactions with our environment can be predicted in advance. For example, we may be provided with accumulating perceptual evidence that will lead to a motor action. In this situation, the brain has the opportunity to optimize excitability of its neuronal populations (e.g., increase excitability of the motor system) so as to optimize performance. Indeed, many studies (Bertelson and Boons, 1960) have documented increased behavioral performance resulting from prior evidence. According to the first principle of the FBO hypothesis, the brain may readily achieve this purpose by reducing the peak-to-peak amplitude of biased oscillations associated with neuronal populations that are related to the anticipated task, and by increasing it for all other populations. There is plenty of experimental evidence to support this concept (e.g., Bidet-Caulet et al., 2012). Figure 6B (modified from Kubanek et al., 2013) illustrates the relative power (i.e., a function of peak-to-peak amplitude) of an oscillatory signal recorded over sensorimotor cortex in a perceptual decision task, in which subjects were asked to push a button depending on the amount of evidence given by auditory clicks. The power of the modulatory signal is progressively reduced for trials of “high” evidence compared to for trials of “low” perceptual evidence. Thus, this mechanism progressively increases cortical excitability in motor cortex, and clearly demonstrates that cortical excitability of local neuronal populations depends not only on present but also on past events.

It is important to recognize that this optimization of brain function cannot readily be achieved by generating a desired phase relationship between neuronal populations: in the predominant situation in which the timing of task execution is not precisely predictable (e.g., in the example above, it is not exactly clear when the movement will occur), a desired functional relationship between two cortical populations can only be achieved using phase synchrony if oscillations governing two different neuronal populations share the same frequency. This is plausible for populations within a particular cortical system (e.g., the visual system), which may be subserved by the same subcortical nucleus. Indeed, existing experimental evidence for such phase synchrony across populations (Saalmann et al., 2012; Roberts et al., 2013) was derived from data collected within the visual system. At the same time, it is well-known that oscillations in different systems can be produced by different sources, and often have different frequencies (Pineda, 2005). E.g., the frequency of the sensorimotor mu rhythm has been reported to be significantly higher than that of the classical visual alpha rhythm (Storm van Leeuwen et al., 1976). Thus, if the timing of task execution is not known ahead of time, it appears to be difficult if not impossible for the brain to predictively control information flow by achieving constant phase synchrony across such different systems. This suggests that CTC cannot explain the regulation of information flow across wide areas of the brain in such situations.

The situation is opposite if the brain has to process and react to a stimulus that cannot be anticipated, e.g., a loud noise while we are reading. While it is well-known that we can quickly react to such unexpected stimuli (Yantis and Jonides, 1984), such rapid reactions cannot readily be explained by increased excitability that are due to reduction in oscillatory peak-to-peak amplitude, as highest excitability would not be achieved until the oscillation reaches its trough (i.e., up to tens of ms later). Thus, reducing the peak-to-peak amplitude of a biased oscillation would not guarantee that the initial action potential volleys produced by the stimulus would hit excitable neuronal populations in the appropriate sensory regions, and consequently would reduce the ability of the brain to process this stimulus. At the same time, it is well-known that the brain has the ability to reset the phase of oscillatory activity (Brandt, 1997) in response to salient stimuli. With phase-resetting of biased oscillations, the brain could produce oscillatory phase synchrony throughout the respective perceptual system. Thus, it would guarantee that action potential volleys produced by such stimuli would be delivered to excitable neuronal populations throughout that system. While there is evidence for cross-modal phase resetting (Thorne et al., 2011), the degree to which different systems are phase reset by an incoming stimulus may be a critical determinant of the limitations of human performance in sensori-motor behavior. Such phase resetting may even cause subsequent reduction in peak-to-peak amplitude in this perceptual system. Hence, in response to a sudden salient stimulus, the brain may update its ongoing predictions to incorporate the likely case that more salient stimuli will follow the first.

Irrespective of whether an event can or cannot be predicted based on prior evidence, such configurations fundamentally requires the brain to make predictions: in the decision-making example above, the brain must use current and past evidence to make a prediction of the optimal future state of cortical excitability. In the example of a loud noise during reading, the brain must be able to evaluate the likelihood that a particular stimulus occurs given past evidence (e.g., we know that a loud stimulus in a library will produce a stronger cortical response than a loud stimulus in a predictive series of loud stimuli). In other words, the brain must constantly use information from past events to predict the likelihood of a particular stimulus, and adjust cortical excitability as a function of this predicted likelihood. This invokes an image in which the “excitability landscape” across the cortex is constantly being updated using a predictive process.

In summary, the second principle of the FBO hypothesis suggests that variable cortical function is implemented primarily by variable biased oscillations across different cortical populations, and proposes that the variability of the two main parameters of biased oscillations, i.e., oscillatory peak-to-peak amplitude and phase, must be determined by a predictive process. Thus, predictive biased oscillations can form the basis for a simple, general, and physiologically grounded model of variable cortical function.

## Predictions

The FBO hypothesis generates a number of testable predictions. The first principle of the FBO hypothesis predicts: (1) that for most if not all locations in the cortex that are modulated by oscillatory activity, oscillatory activity has a voltage bias that is related to oscillatory power; (2) that the instantaneous voltage of biased oscillations is a better predictor of cortical excitability (e.g., as assessed by action potential firing probability or by the magnitude of broadband gamma amplitude^4^) than is oscillatory power or phase; (3) that amplitude variations in biased oscillatory signals can explain a fraction of the variance of slow time-domain signals (such as the BP), of ERPs, and of more recent observations (in particular amplitude-amplitude coupling or PAC that involves frequencies < 4 Hz); and (4) common evoked responses (ERPs) that are routinely detected in EEG/MEG may not be detectable in LFP or ECoG signals, because ERPs represent at least in part the spatially superimposed time-domain voltage changes associated with a temporal sequence of oscillatory power adjustments that are the consequence of a stimulus.

The second principle of the FBO predicts: (1) that variable routing of information flow through a physical network depends primarily on the cortical excitability (indexed by biased oscillations) of the receiving neuronal population; (2) that the peak-to-peak amplitude of a biased oscillation is produced by a prediction of the likelihood that the corresponding neuronal population is related to the task; (3) that the phase of a cortical oscillation is adjusted as a function of a prediction of the likelihood of a sensory stimulus; (4) that differential oscillatory activity should be present not only across different systems (e.g., visual vs. motor), but also within a particular system; and (5) that task execution (rather than predictive network modulation) should always be accompanied by non-oscillatory broadband gamma activity.

Testing these predictions requires careful consideration of several technical issues. First, any particular cortical population may be under simultaneous and superimposing modulatory influence by different oscillations (e.g., Hughes and Crunelli, 2007; Jacobs et al., 2007). Second, the raw voltage potential may be influenced by non-oscillatory activity (e.g., voltage shifts created by ionic currents). Third, voltage is not an absolute but a relative measurement. Thus, an experimentally measured voltage bias may be of varying magnitude or even polarity depending on sensor modality and source of referencing. Fourth, with present signal acquisition hardware, it is difficult to achieve similar signal-to-noise characteristics across all relevant signal frequencies (i.e., DC to high gamma). Fifth, oscillatory modulation is likely to be spatially fine-grained, and hence may be subjected to spatial summation, which will impede its proper characterization using EEG or MEG. Thus, testing these predictions may benefit greatly from, and will likely require, intracranial or intracortical recordings.

## Further research

The FBO hypothesis provides a proposal for two general mechanisms that can support dynamic cortical function. Its main predictions listed above can now readily be tested in future experimental research. In addition, there are several important questions that remain to be answered.

1. Inline with previous findings, this paper suggests that there is an asymmetric distribution of peak and trough amplitudes. The specific characteristics of this asymmetry are currently unclear.
2. Is cortical excitability influenced by factors other than instantaneous voltage?
3. Other than instantaneous cortical excitability, which factors (such as amplitude or temporal distribution) of input to a given region determine cortical excitation?
4. Why is cortical excitability established using repetitively pulsed inhibition (i.e., oscillatory activity) rather than using a continuous process? I speculate that repetitive inhibition may be more metabolically efficient than continuous inhibition, and may be equally effective.
5. The second principle of the FBO hypothesis explains how the brain may predictively modulate cortical function. It does not attempt to answer several important corresponding questions:
  - How does the brain generate predictive models of optimal cortical excitability?
  - How does the brain use sensory inputs resulting from particular behaviors to change the parameters of these predictive models to optimize future behaviors?
  - The predictive processes described in the FBO hypothesis essentially bias cortical processing toward those neural populations that are task-related. It does not elucidate the nature of the cortical activations that actually execute the tasks (i.e., primarily detected using action potential firing rates or broadband gamma amplitude). The relationship between these two processes is important, because they lead to different predictions about measurements. As an example, according to the FBO hypothesis, presentation of multiple sensory stimuli will lead to an increase in cortical excitability in the regions corresponding to the particular sensory domain. Thus, subsequent stimuli should result in augmented cortical responses. However, many experiments have shown that repeated stimulation can result in decreased responses, a phenomenon called repetition suppression (Baldeweg, 2006). This phenomenon may be explained by the concept of *predictive coding* (Friston, 2010; Clark, 2013), which postulates that coding of information in the brain at least in part represents the discrepancy between a prediction of a sensory stimulus and the actual stimulus. In summary, these two concepts may lead to completely opposite experimental results. Future research is necessary to establish the interplay between these two phenomena.

### Conflict of interest statement

The author declares that the research was conducted in the absence of any commercial or financial relationships that could be construed as a potential conflict of interest.

## Footnotes

^1^It is important to recognize that the polarity of these voltage changes depends on the recording configuration, and thus may be positive or negative.

^2^A later article (Nikulin et al., 2010) came to a somewhat different conclusion.

^3^The amplitude envelope of an oscillation is the square root of the power envelope. While they are different mathematically, for the purposes of the arguments presented here, they can be used interchangeably.

^4^Broadband gamma amplitude is often computed by determining the analytic amplitude of ECoG/LFP signals in a high (e.g., 70–170 Hz) frequency band. Broadband gamma activity has been suggested by an increasing number of studies to reflect the average firing rate of neuronal populations close to the electrode (Manning et al., 2009; Miller et al., 2009; Ray and Maunsell, 2011)
